# Self-Powered Smart Beehive Monitoring and Control System (SBMaCS) †

**DOI:** 10.3390/s21103522

**Published:** 2021-05-19

**Authors:** Elias Ntawuzumunsi, Santhi Kumaran, Louis Sibomana

**Affiliations:** 1 African Center of Excellence in Internet of Things (ACEIoT), College of Science and Technology, University of Rwanda, KN Street Nyarugenge, Kigali 3900, Rwanda; 2 Department of Computer Engineering, School of ICT, Copperbelt University, Kitwe 21692, Zambia; santhi.kr@cbu.ac.zm; 3 National Council of Science and Technology (NCST), Kigali 2285, Rwanda; sibomana@ncst.gov.rw

**Keywords:** Smart Beehive Monitoring and Control System (SBMaCS), energy harvesting, beekeeping, smart beehive, piezoelectric transducer, bees’ vibration

## Abstract

Beekeeping in Africa has been practiced for many years through successive generations and along inherited patterns. Beekeepers continue to face challenges in accessing consistent and business-driven markets for their bee products. In addition, the honeybee populations are decreasing due to colony collapse disorder (CCD), fire, loss of bees in swarming, honey buggers and other animals, moths, starvation, cold weather, and Varoa mites. The main issues are related to un-controlled temperature, humidity, and traditional management of beekeeping. These challenges result in low production of honey and colony losses. The control of the environmental conditions within and surrounding the beehives are not available to beekeepers due to the lack of monitoring systems. A Smart Beehive System using Internet of Things (IoT) technology would allow beekeepers to keep track of the amount of honey created in their hives and bee colonies even when they are far from their hives, through mobile phones, which would curtail the challenges currently faced by the beekeepers. However, there are challenges in the design of energy-efficient embedded electronic devices for IoT. A promising solution is to provide energy autonomy to the IoT nodes that will harvest residual energy from ambient sources, such as motion, vibrations, light, or heat. This paper proposes a Self-Powered Smart Beehive Monitoring and Control System (SBMaCS) using IoT to support remote follow-up and control, enhancing bee colonies’ security and thus increasing the honey productivity. First, we develop the SBMaCS hardware prototype interconnecting various sensors, such as temperature sensor, humidity sensor, piezoelectric transducer—which will work as a weight sensor—motion sensor, and flame sensor. Second, we introduce energy harvesting models to self-power the SBMaCS by analyzing the (i) energy harvested from adult bees’ vibrations, (ii) energy harvesting through the piezoelectric transducer, and (iii) radio frequency energy harvesting. Third, we develop a mobile phone application that interacts with the SBMaCS hardware to monitor and control the various parameters related to the beehives. Finally, the SBMaCS PCB layout is also designed. SBMaCS will help beekeepers to successfully monitor and control some important smart beekeeping activities wherever they are using their mobile phone application.

## 1. Introduction

Globally, beekeeping has been practiced for many years and many organizations have been involved in supporting beekeeping activities [1], but still, the sector remains underdeveloped. The activity has basically been traditional and non-commercial in nature. Further, beekeepers face challenges in accessing consistent and business-driven markets for their bee products even though bees are a source of money, food, medicine, and help us to preserve the environment. Beekeepers have been facing various challenges due to the environmental conditions surrounding the beehives and within the beehives, such as un-controlled temperature, humidity, and traditional management of beekeeping [2]. These challenges result in low production of honey and colony losses. In addition, the honeybee populations are decreasing due to colony collapse disorder (CCD) [3]. Furthermore, the information on this sector remains scattered, with most of the information available amongst various sector stakeholders merely assumptions due to the lack of a monitoring and evaluation system and sharing of information.

In order to improve the above situation, there is a need to develop a platform through which we can monitor, collect, and analyze the conditions within the bee colonies, and emerging technology such as Internet of Things (IoT) can be used for remote monitoring.

Traditionally, batteries are used as the electrical energy source to power wireless sensors and other embedded electronic devices in a smart system. The limited lifespan of batteries is one of the main factors constraining the performance of such systems. Hence, they are not a long-term viable source of energy. Moreover, they are expensive to maintain. Energy harvesting is the most promising way of overcoming the challenges currently presented by finite life power sources such as batteries. The process of energy harvesting involves the harnessing of ambient energy from within the vicinity of the sensor device and converting this energy into usable electrical energy. Compared to batteries, energy harvesting presents a potentially infinite source of energy for powering wireless sensor devices and embedded electronics in general. However, what is new with energy harvesting technology is how to design and implement efficient energy harvesting capabilities into modern embedded systems while satisfying all their criteria. For any energy harvesting system to be attractive, it should allow miniaturization and integration using the present micro-electro-mechanical systems (MEMS) technology, otherwise it is not useful for embedded system applications. While solar energy harvesting is a fairly established technology, it is not the best choice for mobile, implantable, and embedded electronics where solar energy is not accessible [4]. Mechanical energy in the form of ambient vibrations, fluid flow, machine rotations, and biomotion presents a source of energy that is widely available at all times. Piezoelectric materials can be used to harvest this energy since they have the unique ability of converting mechanical strain energy into useful electrical energy. Piezoelectric energy harvesting devices—in the form of MEMS generators or nanogenerators—are a novel technology that is a reliable alternative energy source for powering smart bees farming technology. The main contributions of this paper are listed as follows:

1. A smart beehive multi-sensing circuit board will be developed integrating various sensors, such as temperature sensor, electric fan and thermo-electronic heater, digital camera, and global positioning system (GPS) to track the location of the hive.
2. A mobile application interfacing with the smart beehive, allowing beekeepers to keep track of their bee colony and honey even when they are far from the hives through an application in their mobile phones or through an automated system connected on the Cloud.
3. Different parameters, such as temperature, humidity, weight, gas and flame, will be monitored and regulated remotely through a mobile phone or through an automated system connected on the Cloud. The security of the bees in the hive will also be monitored remotely.
4. Generation of energy based on different energy harvesting technologies, such as piezoelectric energy harvesting from the force of the hive applied to a piezoelectric transducer, from bees’ vibration, and electromagnetic wave and energy harvesting from the surrounding environment where the hive is located.

The proposed new system will achieve the following five objectives:

- To design and develop an IoT-based SBMaCS using multi-sensors.
- To provide beekeepers real-time updates on their mobile phones about the real life of bees inside the hive and about the environment surrounding the hive.
- To provide an automatic control of temperature and humidity of the hive for the purpose of enhancing the honey production.
- To design a self-powered model for SBMaCS based on different energy harvesting technologies.

The rest of this paper is organized as follows. Section 2 discusses related research works. Section 3 presents the architecture and energy consumption of the proposed system. The energy harvesting model for the self-powered system is discussed in Section 4. Section 5 presents the lab experiments conducted and simulation results, and finally, we end with the conclusion in Section 6.

## 2. Related Works

The colonies’ remote monitoring system has been discussed by many researchers, but there are still many gaps. Bee colonies’ monitoring started in the 11th century [5]. Recently, with the advancement in electronics, authors in [6] developed a hive monitoring system to monitor the available temperature and humidity. They detect when the hive is blown away and can access data through any web browser [7,8,9,10]. In [11], control of the beehive parameters remotely was not dealt with. Many other important parameters, such us weight of the honey inside the hive and gas produced by bees in the hive, were neither accessed nor remotely controlled. However, the system presented in [12] does not measure the periods where the bees are hungry or dying.

In [13], the authors presented a recording paper-based beekeeping information system using pen and paper. The limitation of such recording system is the need to deliver messages to distant places to share information, and slice and splice of the data to spot trends. Further, the authors in [14] focused on hive tracking through a mobile application to track the progress of hives and keep tabs on the bees, but still there is a gap as the information is not readily accessible and shareable. The authors in [15] presented a beehive monitoring and tracking system using a weight sensor, where a significant number of parameters were gathered, especially the data related to the status of honey inside the hive. This data is collected and uploaded to a web-based service, without using it for control purposes. 

Thus, there is a need to introduce an effective beehive monitoring system to track, make available, and provide all related information to beekeepers on a real-time basis. Monitoring beehives is an effective and easy way of tracking the problems related to beekeeping so that a proper action could be taken on time. For example, decreasing the bee population results in beekeepers losing their money and a declined production of food, which has an indirect impact on everyone [16]. Our proposed SBHMAS is an integrated system of intelligent hives, where data gathered from beehives are used efficiently to remotely control the environment surrounding the hives and inform beekeepers through mobile phone or Cloud-based systems. Moreover, the work in [17] predicted that the world will need 30 TW of energy resources around the year 2050 to maintain economic growth of the countries. This implies continuous growth of energy demand on the market. Besides, the ubiquitous presence of radio and TV bands, cell phones and personal communications devices, a myriad of Wi-Fi stations, Bluetooth gadgets, and remote emitters and detectors produce a non-negligible amount of EM energy flowing around us [18]. The authors in [19] presented Arnia hive monitoring technology to monitor some parameters of the apiary, such as temperature and humidity, and the system is powered with solar panels. This solar energy source has a limitation in that most of the beehives are located in remote areas surrounded by trees, which would result in difficulty for the sunlight to reach the solar panel. To address this, the work in [20] has shown that harvesting energy from bees’ motions can produce a kind of mechanical vibration which is converted into electrical energy. However, once the colony dies, the system will stop working since the power source (bee motion) is no longer available. However, in the case of bees, as they are always vibrating for different purposes, such as, for example, when they dehydrate the honey with ventilation, when they heat the hives in winter, while they refresh it in summer through ventilation and the use of water, when they are going to seal the honey, when they are pushing the excess heat out of the hive, when they are generating heat to increase the level of heat in the hive, and when they are communicating (they use dancing method), this type of energy harvesting from motion is a way of recycling the energy to be used again [21,22,23]. 

The proposed SBMaCS is a self-powered system based on different energy harvesting technologies, such as piezoelectric, bees’ vibration, and electromagnetic sources. Through the self-powered SBHMAS tool along with efficient energy harvesting models for supplying energy to the various components, beekeepers located in different areas will be able to remotely monitor the surrounding environment of the hive through their mobile phones or a dynamic platform connected to the Cloud. To maintain a good life condition of bees in the hive, different IoT devices are interconnected dynamically and they consume the energy harvested by the system.

## 3. Architecture of Smart Beehive Monitoring and Control System (SBMaCS) 

**Figure 1:**
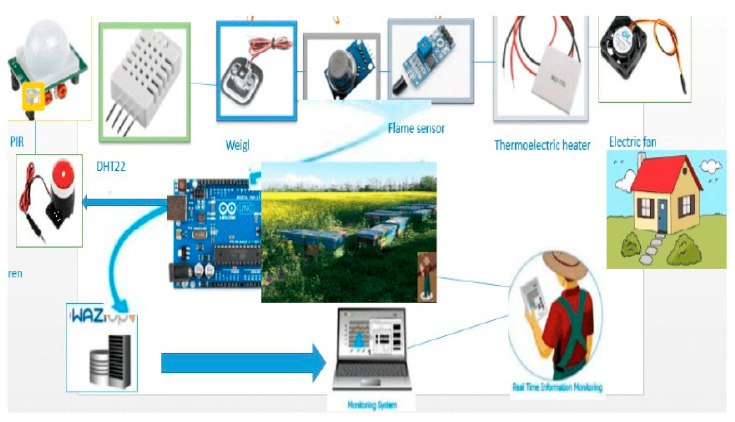
SBMaCS architecture.

The integrated SBMaCS for remote monitoring and control is shown in Figure 1. These components include a microcontroller, GSM module, LoRa gateway, and different sensors, such as a humidity sensor, weight sensor, flame sensor, PIR sensor, temperature sensor, and an electronic fan, electromagnetic heat, and a digital camera. The control and communication mechanisms are aided by a software component developed by programming the microcontroller.

**Figure 2:**
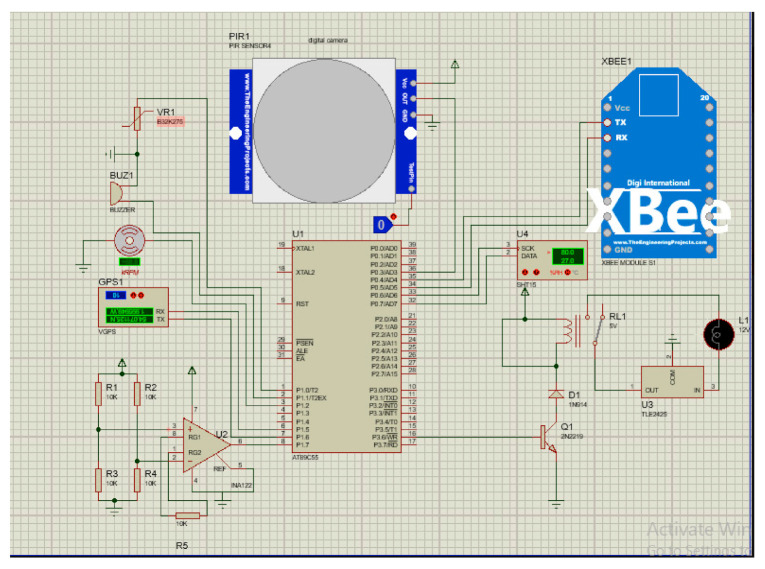
Schematic design of SBMaCS.

SBMaCS has a liquid crystal display on the hive displaying all information of the hives, which helps beekeepers to maintain remote control of the beehives. The temperature of the hive is an important factor to be maintained. An automatic controlled thermostat that works in the optimum range (32–36 °C) is installed so that bees work well [24], and bees always need this temperature when they have larvae. The weight of the hive increases gradually when the queen is working properly. If the weight is decreasing, the bees have a shortage of food or the queen is not working properly. We set a threshold minimum weight and a threshold maximum which give an SMS alert to the farmers when the weight reaches these thresholds. The flame sensor gives an alert to the beekeeper when a forest fire or any kind of smoke is detected. For protecting the bees from the varoa mites, we need to control the humidity inside the hive based on a minimum threshold of humidity. Furthermore, an electric fan is used to help ventilate the hive and control the humidity. Image sensors were installed to detect birds or moths entering the hive during propolis production [25,26]. If they enter, the beekeeper gets a signal from the hive on his/her mobile phone. An alarm will be received on the phone of the beekeeper if there is problem such as displacement of the hive or other problems related to the security of the apiary. The weight sensor will indicate if the hive is blown away from the stand. These sensors help beekeepers to gather information about the hive and automatically send information to the owner of the apiary. In addition, there are also digital cameras for ensuring security of the apiary. LoRa technology is used to facilitate the transmission of data over a long distance. The schematic design is shown in Figure 2. 

The above circuit shows how different components of SBMaCS using multi-sensors were connected on a microcontroller.

**Figure 3:**
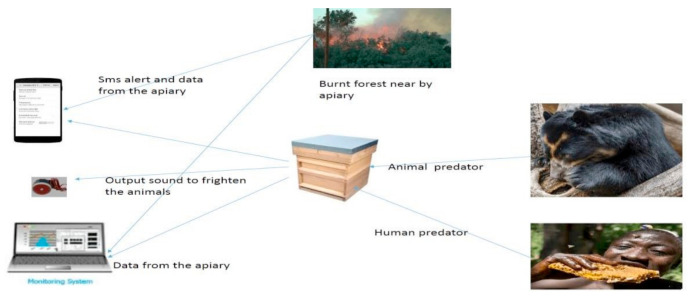
SBMaCS system interaction.

Figure 2 shows the circuit diagram of SBMaCS internal components’ connections, while Figure 3 shows how the SBMaCS system is used to protect the apiary, where there are some external features such as a flame sensor to inform the beekeeper if there is fire around the apiary to help save the bees before being burnt. There is also an external air quality sensor which will help to identify the environment in which the bees are working. The internal gas and external gas need to be compared to identify the health condition of the bees. As for the quality of the air around the apiary, the exhaust gases have a serious effect on the working condition of bees, and bees bring these gases into honey, which affects the health of the people who use it. The digital siren is used to threaten the animals which come to harvest honey, and at the same time, the apiary’s owner receives an alert message that there is insecurity in the apiary.

### Energy Consumption by SBMaCS

Energy consumed by SBMaCS was calculated based on the number of sensors integrated on the network of the whole system.

(i) DHT11 is a temperature and humidity sensor used for measuring the temperature and humidity to monitor the heating of the system. It needs to always be on to measure the temperature and humidity so it can turn on thermoelectric heating and the digital fan to the set conditions. The power consumption of DHT11 is specified in Table 1.
(ii) The fan is the transducer integrated for the purpose of replacing the work of vibrating done by bees, the power specifications of the fan are:
  - The power dissipation is 585 Mw, the output voltage is 22 V, and the output current is 700 Na.
(iii) Flame sensor is a module applied to the fire detection system for the alarm system. It is integrated on the smart bee farming technology and monitoring system to detect the outbreak of fire around the apiary to protect the beehives against fire and intruders who can try to burn beehives. The operating voltage is 3.3–5.3 V
(iv) MQ135 is gas sensor which applies SnO_2_. It has a lower conductivity in the clear air as a gas-sensing material. In an atmosphere where there may be polluting gas, the conductivity of the gas sensor rises along with the increased concentration of the polluting gas. It performs good detection of smoke and other harmful gases, and is especially sensitive to ammonia, sulfide, and benzene steam. The power consumption plan of the module is the following:
  - Operating voltage is 2.5–5.0 V
  - Heater consumption is ≤900 mW
  - Sensing resistance is 2–20 KΩ (in 100 ppm NH3)
  - Load resistance is adjustable
  - Heater voltage is 5.0 ± 0.2 V AC or DC
  - Total power of SBHMAS

Table 2 shows the energy of different sensors during active, standby, peripheral, data log, and transmission currents mode of each component. From the energies in the Table 2, a model of energy harvesting is calculated based on rescheduling of each sensor shown in Table 2.

From the rescheduling of sensors cited in Table 2, sensors which are scheduled will be equal to:

(1) $$K=N-\left(H+S\right)$$

where *N* is the total number of components connected to the microcontroller, and

(2) H+S

stands for the number of all unscheduled components. The time of each scheduled sensor is equal to:

(3) $$T=\frac{D}{K^{\prime }}$$

where D is the period of the complete cycle of the whole system, and *k*′ is the number of sensors that are scheduled. The ratio of time for each transducer (*R*) is *T*, equal to 100%, and then the time required by the sensors to be in standby mode will be equal to:

(4) $$T_{S}=\frac{R}{100}*90$$

The time required by the sensors to be in active mode is equal to:

(5) $$T_{a}=\frac{R}{100}*90$$

## 4. Energy Harvesting Model for Self-Powering the SBMaCS 

There are potential ways available to harvest energy from the ambient environment, including solar energy, wind power, radio frequency energy, vibration energy, and thermal energy [27]. Each of them has its own potential advantages and disadvantages depending on the application and the location of the wireless sensor network. In this article, we propose to use three energy harvesting technologies for self-powering SBMaCS: piezoelectric transducer energy harvesting, electromagnetic energy harvesting, and energy harvesting from bees’ vibration. 

### 4.1. Piezoelectric Transducer Energy Harvesting Model

Piezoelectric materials have a significant property whereby whenever pressure is applied on them, they become mechanically strained and electrically polarized. Due to the pressure, an electric field is generated, and voltage is induced [28]. This effect is known as the direct piezoelectric effect. The degree of polarization is directly proportional to the applied pressure or strain. Furthermore, the amount of applied pressure is directly proportional to the voltage. Conversely, the induced voltage or electric field causes a change in the amount of strain and the material is mechanically deformed [29]. The transducer (piezoelectric) is used to generate the power with the use of pressure from the hive and the vibration from the bees inside the hive. The following are the two methods used in order to harvest energy:

- Energy harvesting with the use of kinetic energy or pressure

**Figure 4:**
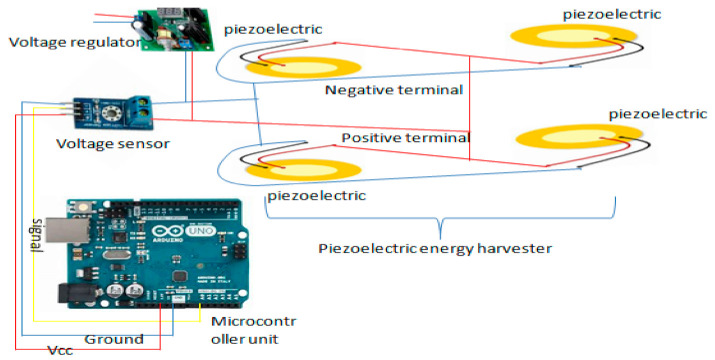
Piezoelectric energy harvester circuit.

The energy generated (voltage generated) from piezoelectric ceramic materials depends on the following parameters: dimensions of the piezoelectric material, force exerted on the material, level of deformation, loss of load, piezoelectric constant, resistance of piezoelectric transducers, static capacitance of the piezoelectric material, and type of piezoelectric material [30,31,32,33]. Figure 4 describes how power is generated using the piezoelectric energy harvester.

As it is shown in Figure 4, the piezoelectric energy harvester is one of the energy harvesting technologies where energy should be harvested through the serial connection of piezoelectric transducer. The design allows us to measure the weight of the hives while at the same time generating the energy required to supply the system. The generated voltage is sent to the voltage regulator to match the voltage with the load, and it is sent to the voltage sensor for helping the measurement of the force exerted on the piezoelectric power generator; in this case, we can know the voltage generated by the piezoelectric transducer through the following mathematical calculation:

(6) W=V∗I

where W stands for the power generated in watts, V stands for the voltage generated in volts by the piezoelectric power generator, and I stands for the current in amperes. The force deformation or pressure implies that the power generated depends on the weight applied on the piezoelectric ceramic:

(7) F=m∗g

where F is the weight or force of the hive, m is the mass of the object, g (approximately equal to 9.81 m/s^2^) is the acceleration or the force of gravity, and the pressure is approximately equal the ratio of force and area:

(8) $$P=\frac{F}{A}$$

where P stands for pressure and A stands for the area of the ceramic. Equation (8) allows us to calculate the area of the piezoelectric as follows:

(9) $$A=\frac{d^{2}*\pi }{4}$$

where *d* stands for the radius of piezoelectric transducer.

Combining Equations (8) and (9), it leads us to the following formula:

(10) $$A=\frac{F}{\frac{d^{2}\;*\;\pi }{4}}$$

The theoretical bases present the following formula for the voltage generation:

(11) $$V=-g_{33}*h*\frac{F}{\frac{d^{2}\;*\;\pi }{4}}=g_{33}*h*p$$

where h is the height of the ceramic disk, and $g_{33}$ is the piezoelectric constant. From the Formula (11), we see that the voltage generated especially depends on two parameters: mass exposed on the ceramic disk and the area at which the force is exposed by the force when other parameters are kept constant. From Formula (11), we achieve energy for self-powering smart beehive mobile technology. For making the piezoelectric transducer more energy-efficient, we connect more parallel circuits, and from Formula (11), the weight of the hive is calculated as follows:

(12) $$F=\frac{V*\frac{d^{2}\;*\;\pi }{4}}{-g_{33}*h}$$

From Formula (12), we deduce that the force from the hive applied to the piezoelectric transducer connected in series generates energy. This means that there is a mechanical deformation from the force of the hive that changes into electrical power through piezoelectric transducers.

b. Energy harvesting model from the bees’ vibration

**Figure 5:**
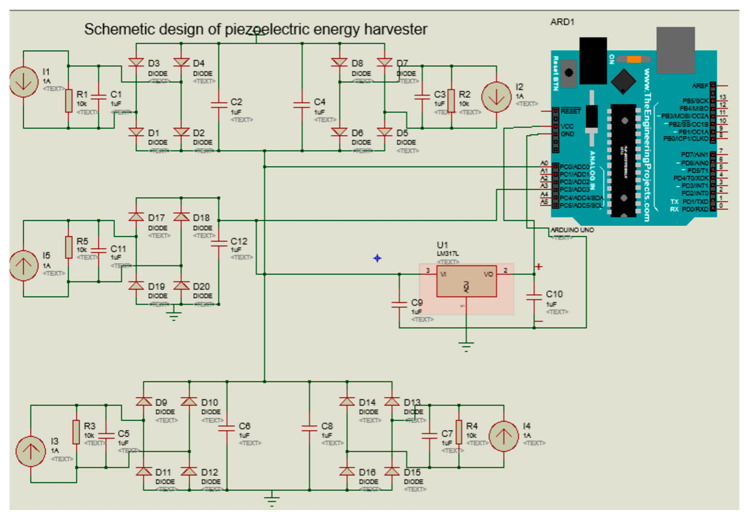
Schematic diagram of piezoelectric energy harvester based on transducer and bees’ vibration.

Piezoelectric energy harvesting has shown the potential to convert insects’ mechanical vibrations into constant electrical energy output [34,35]. Honeybee wing beat rates vary between 208 and 277 Hz depending on physiological differences such as temperature, humidity, and tiredness [36]. This difference renders the resonant energy harvesting approach unrealistic as the vibrations will not match the resonant frequency of the harvester, which could result in the generated power being orders of magnitude lower than resonance. Inside the hive when the bees are waggling to give a signal to their follower bees, the vibration creates a sound with a high frequency within the range of 10 to more than 500 Hz, and the SBMaCS has a digital fan which turns on when the heat reaches the set condition [37,38]. For this frequency, we designed a model to change the bees vibrations into an electrical energy, and the energy harvested is followed with the well-designed circuit to boost the power to fit the designed network (SBMaCS) (Figure 5). The fan consumes a huge amount of energy to maintain the health condition of the hive, and to optimize the energy consumed by the system, we propose to use the vibration energy harvester to power the system. The following formula describes how the vibration energy harvester works:

(13) $$P=\frac{m\varsigma y^{2}(\frac{\omega }{\omega_{n}})\omega^{3}}{{\left[1-{\left(\frac{\omega }{\omega_{n}}\right)}^{2}\right]}^{2}+{\left[2Z\frac{\omega }{\omega_{n}}\right]}^{2}}$$

where ς stands for damping ratio: $\varsigma =q/{\left(2m\;{*\;\omega }_{n}\right)}_{}\omega_{n}$, y is the resonant frequency and the amplitude of the vibration, and ω is the excitation frequency. The maximum output power occurs at the resonant frequency of the generator if ω is equal to $\omega_{n}$ for the system without damping:

(14) $$p_{max}=p_{\left(\omega_{n}\right)}=\frac{my^{2}\omega_{n^{3}}}{4\varsigma }\;\;p_{max}=\frac{mA^{2}}{4{\varsigma \omega }_{n}}$$

where A is excitation acceleration magnitude, A = ω*y*. From the formula of equivalent stiffness:

(15) $$K_{eq}=\frac{3EI}{L^{3}}$$

where *E* is the equivalent elastic modulus, I is the equivalent rational inertia, and *L* is the effective length of the piezoelectric. We can finally see that the resonant frequency is:

(16) $$f_{n}=\frac{\omega_{n}}{2\pi }=\frac{1}{2\pi }\frac{\sqrt{3EI}}{(m+\frac{33}{140}m_{s})L_{3}}$$

where $m_{s}$ is the mass of the piezoelectric transducer, and *L* is the effective length of piezoelectric transducer.

The resulting error most often arises from the imbalance of the body exerting the weight on the piezoelectric disk. To minimize this, we placed the piezoelectric disk in the four corners of the hive to balance the weight of the hive on the disk. Four piezoelectric transducers are put in place, which are connected in parallel to increase the energy harvested, and another vibration energy harvester is connected to the system which is also connected in parallel to the other piezoelectric transducers. Transducers are connected not only to produce power but also to monitor the health of the bees, which is the reason why they are connected to arduino for reading the output. The voltage detector is employed to read the available weight of the hive and the vibration energy harvester will work as an acoustic sensor in the case of monitoring. Then, the harvested power is sent to the energy regulator system.

### 4.2. Electromagnetic Energy Harvesting

Electromagnetic energy is usually captured from the ambient radio frequency (RF) sources, which generate high electromagnetic fields, like TV broadcast stations, radar stations, Wi-Fi routers, Bluetooth, global system for mobile communications (GSM), and other communication networks. RF energy harvesting devices can convert electromagnetic energy into a useful direct current (DC) voltage to power low-power consumer electronics and WSNs [39]. Radio waves reach an antenna and cause a changing potential difference across its length. This potential difference and hence the induced RF energy is captured by the RF to DC converter. The DC energy is stored temporally in a capacitor and then used to power the devices.

**Figure 6:**
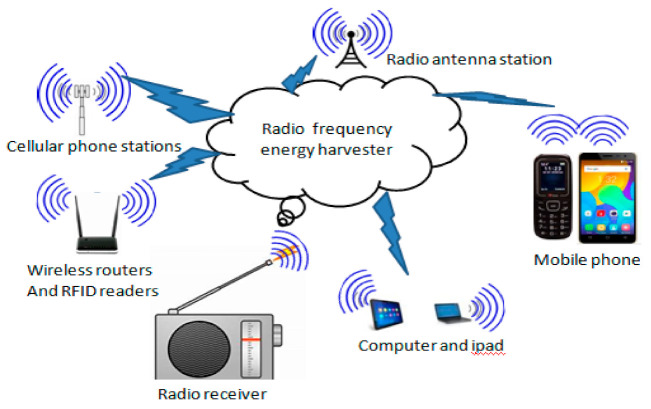
Structure of RF energy harvesters.

A basic RF energy system (Figure 6) consists of a receiving antenna, a matching circuit, a rectifier, and a power management system. The antenna captures the electromagnetic waves and then the matching circuit is used to induce maximum power by using coil and capacitors, and the rectifier then converts the alternating current (AC) signal to a direct current (DC). The antenna in RF energy harvesting systems is investigated in the receiver side for receiving EM waves from the various ambient RF sources, which are broadly available in the surroundings, such as digital TV broadcasting (500 MHz band), mobile phone services (UHF-band downlink), and several other wireless systems [40].

The average power received by the antenna (Pav) depends on the power density (S) and the antenna effective area (Ae):

Pav = S × Ae (17)

The received power (Pr) by the antenna placed at a distance, R, from the transmitter antenna can be expressed as:

(18) $$\mathrm{Pr}=\mathrm{Pt}\;\mathrm{Gt}\;\mathrm{Gr}\;(\frac{c}{2\Pi f}{)}^{2}\;(\frac{1}{R}){\;}^{N}\;e^{-\alpha R\;}$$

where Pt is the power transmitted or the input power to the transmitter antenna, and Gt and Gr are, respectively, the gains of transmitting and receiving antennas. *λ* = *c*/*f* denotes the wavelength of radiation, where *c* is the velocity of light and *f* is the frequency of the RF signal. α is the effective decay coefficient of air. N denotes the path loss exponent, and N = 2 is for free space. Antenna design for RF energy harvesting is based on two radiators, the main one is a printed dipole radiator and the second one is a loop antenna with a parasitic element [41,42]. The parasitic radiator is suitable for receiving RF power in all directions from the main radiator. We have to use a hexagonal microchip path antenna array that operates at high frequency (such as 915 MHz) in order to receive the maximum possible RF energy.

#### Elctromagnetic Energy Harvesting Model

The RF energy harvesting rate of the sensor node from RF energy source *k* in free space channel is P^K^h, obtained based on the Friis equation, as follows:

(19) PKh=ρ β PS GS GH λ^24ΠDk^2

where, P^K^h is the energy harvesting rate, β is the RF to DC power conversion, ρ is an efficient factor, Ps is the transmitted power, GS is the gain of the transmitter antenna, and λ is the wavelength.

The aggregated RF energy from harvesting rate by the sensor node from the ambient RF sources can be computed as follows:

(20) ∑kεΚPKh=∑kεΚρ β  PS GS GH λ^24ΠDk^2

**Figure 7:**
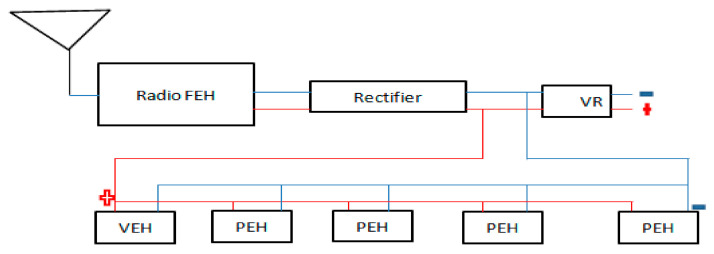
Basic RF energy harvesting system.

**Figure 8:**
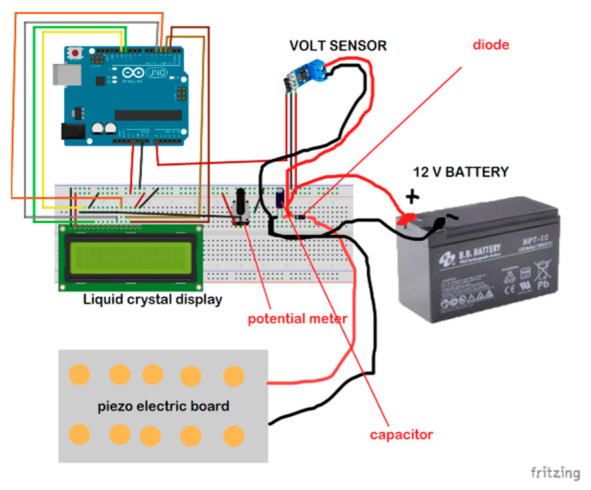

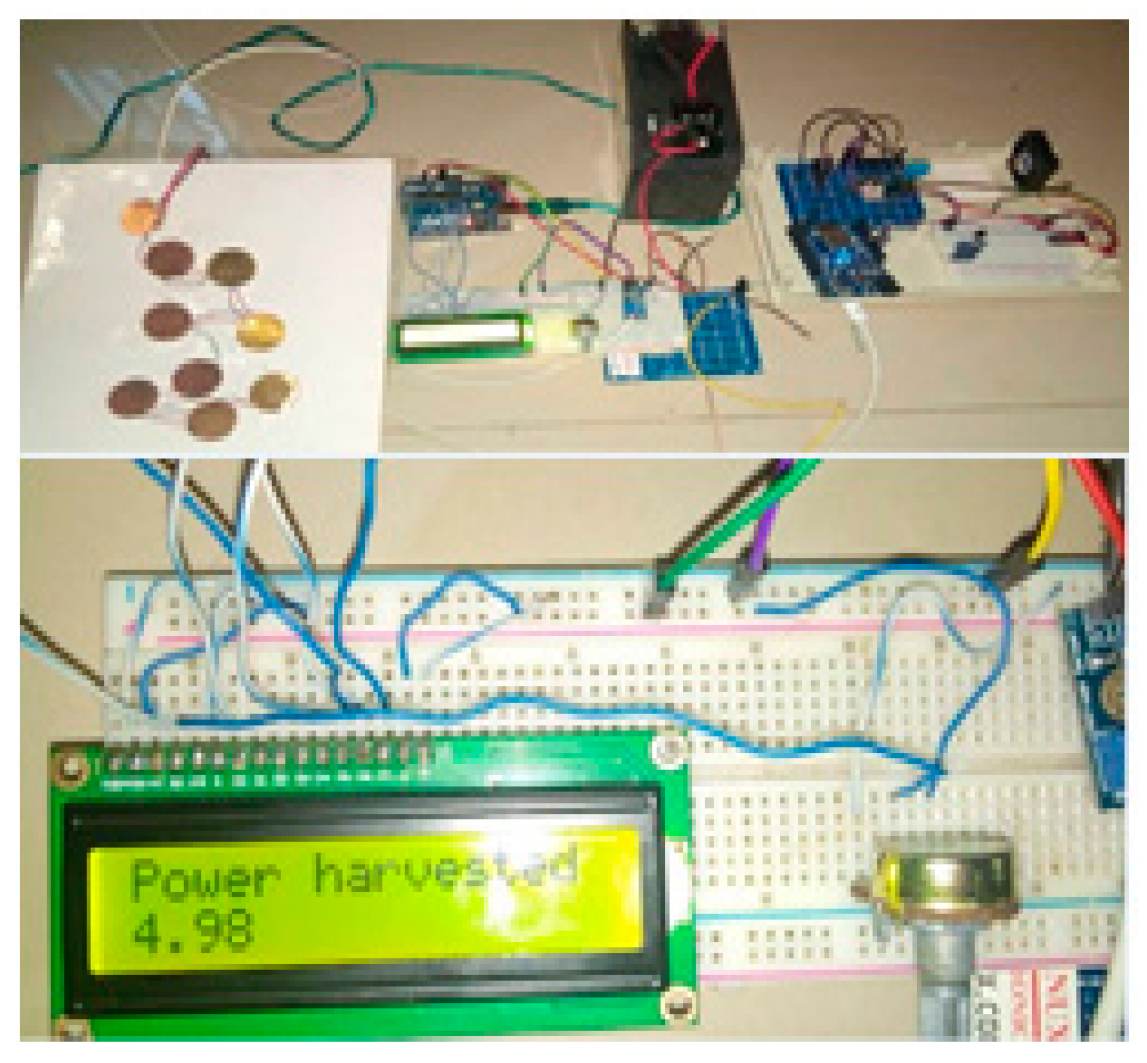
Piezoelectric energy harvesting prototype design.

The basic energy harvesting system is shown in Figure 7. The energy harvesting rate varies significantly depending on the power source and distance. The relationship between received power and distance in the environment of 900 MHz and 2.4 GHz is described in the simulated result shown in Figure 8. As with the development of SBMaCS, RF energy harvesting is one source of energy for self-powering smart beehive monitoring and control systems, however, such RF signals are rarely used in the mechanical systems. The RF sources around machines are usually undesired and thus the potential energy harvested from these RF signals is limited. It is insufficient as a primary power source, but RF energy harvesters can assist other energy generators [43,44]. Additionally, the RF energy harvesting technology still has a bright prospect due to its rapid development and wide application in wireless networks. Therefore, from these three energy harvesting technologies, energy was harvested for self-powering SBMaCS.

## 5. Experiments Conducted and Simulation Results

### 5.1. Experimental Set-Up of Piezoelectric Energy Harvesting Based on the Force of the Hive and Bees’ Vibration

For self-powering the SBMaCS using a piezoelectric harvester, a piezoelectric board was developed for generating some voltage to power the system. The liquid crystal display is added to show the values of voltage harvested from the piezoelectric board, a capacitor temporarily saves the harvested power, and then the diode directs the current into one direction for charging the 12 V battery. The battery requires no other charging means as the piezoelectric alone does it with perfection, and so the system is self-powered. As shown in Figure 8, force due to the weight of the hive is applied to a set of piezoelectric boards divided into two branches of five piezoelectric transducers, and each branch is connected in series. Once the force is exerted on the piezoelectric transducers, there will be a mechanical deformation, and force is transformed into electrical energy through those piezoelectric transducers. Furthermore, the frequency from the bees’ vibration in the hive was also transformed into electrical energy. Therefore, from the experimental results, it was proven that the energy required for self-powering the smart beehive components was generated.

### 5.2. Simulation Results of Energy Harvesting

**Figure 9:**
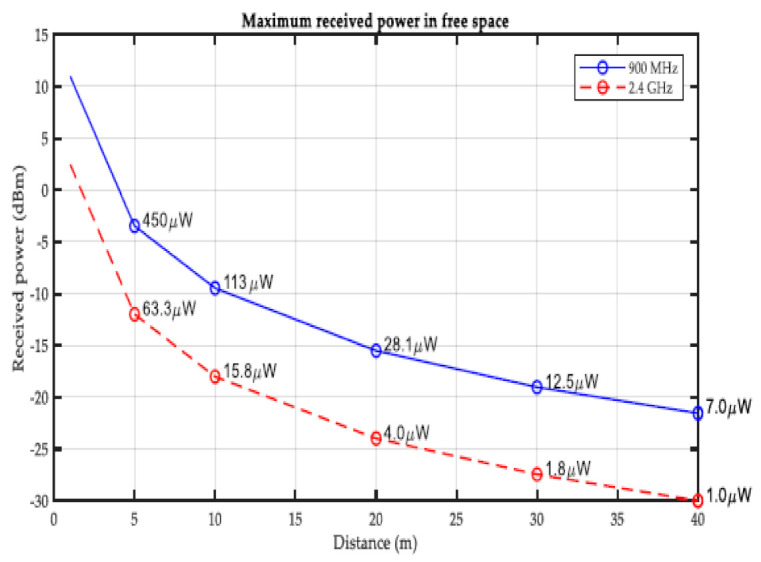
RF energy harvested vs. distance.

**Figure 10:**
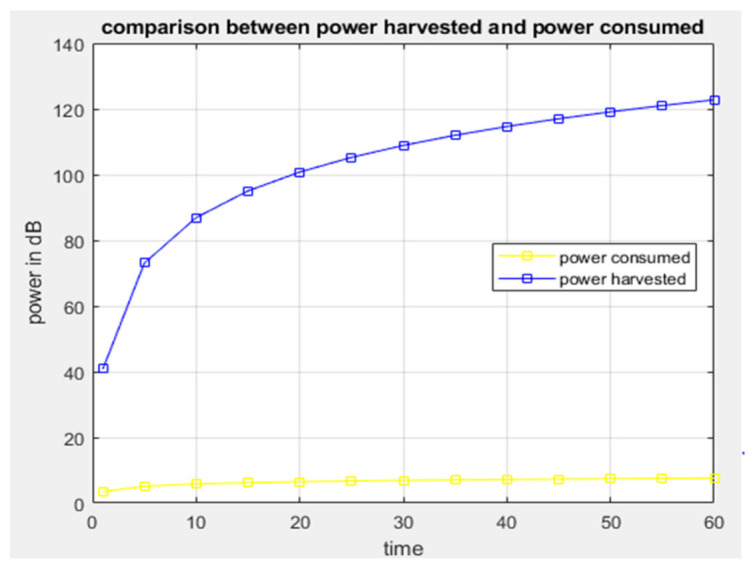
Graph of power harvested vs. power consumed.

**Figure 11:**
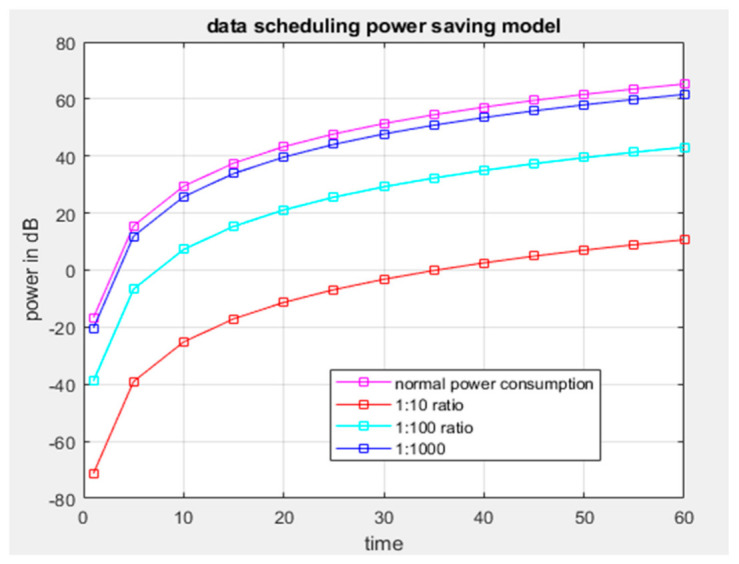
Data scheduling power-saving model.

**Figure 12:**
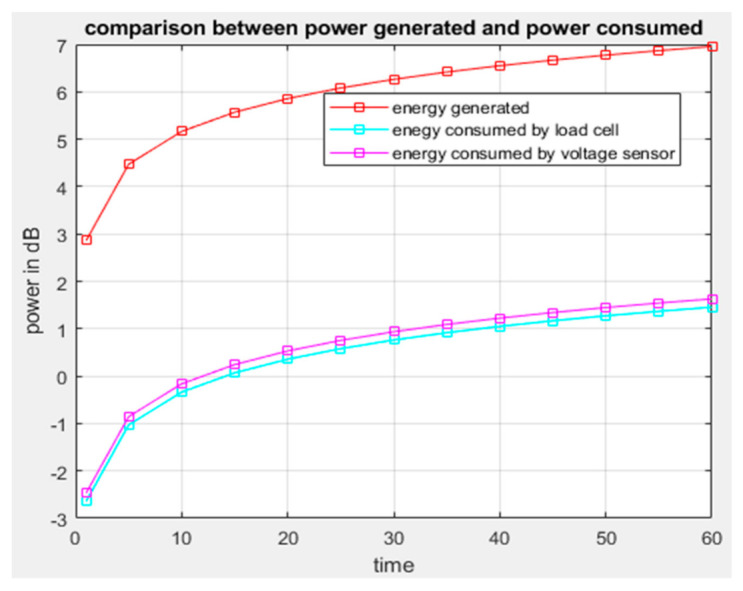
Graph comparing generated power and power consumed.

Results from Figure 9 show that if the distance between a system location and the source of radio frequency power harvested is short, then the power received by the system will be enhanced because the strength of the frequency signal depends on the distance of radio frequency materials surrounding the system. Figure 10 shows the comparison between power generated by the system operation through energy harvesting and consumed by the system at a specific period of time. It is observed that power generated or harvested at any time by the system is very high due to the various harvesting technologies embedded in the system [45]. At each time interval, the system harvests more energy compared to the consumed energy, and the experimental results show that the system harvests sufficient energy for self-powering the system. Figure 11 displays the data scheduling power-saving model, and the graph shows that without data scheduling techniques, a large quantity of power is consumed by the system. It is seen that during data scheduling, an optimum active to standby ratio (ASR) is needed. The experimental results reveal that a 1:10 ASR is required to achieve the maximum power-saving model. The partition of the sensors with respect to time depends on the number of sensors integrated on the system. Furthermore, Figure 12 compares the power generated by the piezoelectric kinetic-based energy harvester and the power consumed by the voltage sensor, which will be used to measure the weight of the hive and the power which is consumed by the load cell.

### 5.3. A Smart Beehive Monitoring and Control System (SBMaCS) Prototype Experimental Set-Up

Different sensors and actuators, such as temperature sensor, electric fan, and thermo-electronic heater, are used to implement the proposed SBMaCS. In addition, the prototype captures stimuli from its environment, including temperature, gas, and weight, to provide real-time output and notifications to the dedicated beekeeper, and these parameters give an idea to the beekeeper of how to control and maintain the optimum temperature inside the hive.

**Figure 13:**
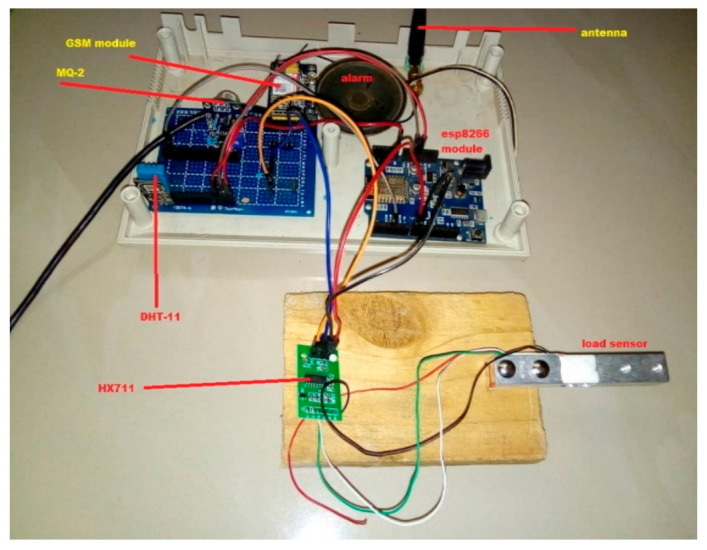
Lab-based experiment of the system.

**Figure 14:**
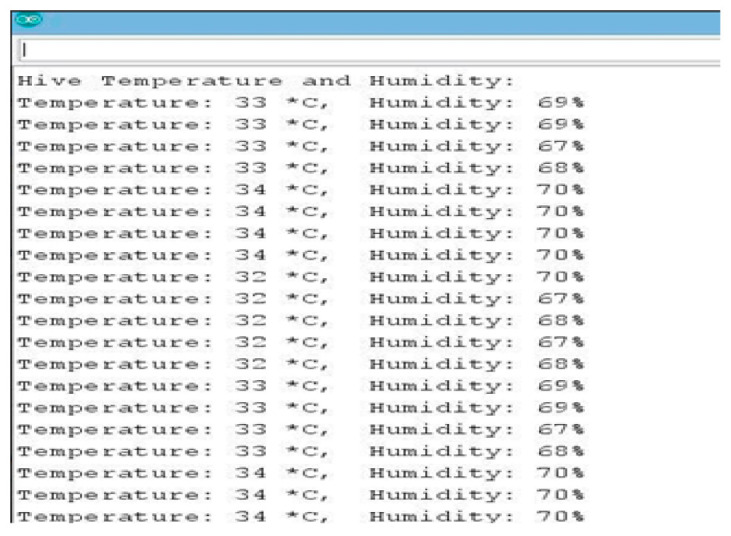
Temperature and humidity results.

As it is shown in Figure 13, the ESP8266 module enhances the performance by making the data accessible to the mobile application by hosting the captured data to a Cloud server, while the GSM module reports events by SMS notification. Figure 14 shows that the set optimum temperature is in the range of 32 and 36 °C, which is the normal range of temperature that bees should have to maintain good health conditions. The fan is set to work automatically if the temperature goes above 32 °C, regulating the temperature to optimum to maintain the humidity inside the hive. Furthermore, it shows that the temperature is maintained in the range of 32 and 36 °C inside the beehive to keep the bees strong enough and to minimize the volume of honey they consume themselves if they have to self-regulate the temperature inside the hive. The thermoelectric heater works in conjunction with the electric fan, regulating the humidity to always be in a range which is suitable for bees to produce more honey and be in better health condition. If it is time for the pre-warming condition, the humidity is increased because high humidity is required for bees to hatch well.

**Figure 15:**
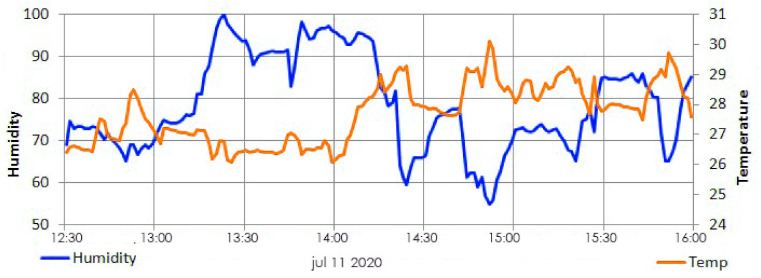
Temperature and humidity relation.

Figure 15 shows that when the system is on for an hour, the humidity inside the beehive decreases between 28.6% and 29%, and this is not the optimum humidity for bees to have better health and to hatch better. When the humidity is much less, bees tries to regulate the condition of the hive, but when it lasts for a long time, they fail to maintain the normal condition. The bee colony regulates humidity by fanning, droplet extrusion, and tongue lashing, and passively by having moisture sinks. This hinders them from working and results in starvation as they spend a lot of time regulating the humidity instead of searching for natural food. In our proposed system, the electric fan and the heater will work in conjunction, alternating themselves, to regulate the humidity and temperature inside the hive, as it is shown in Figure 15.

**Figure 16:**
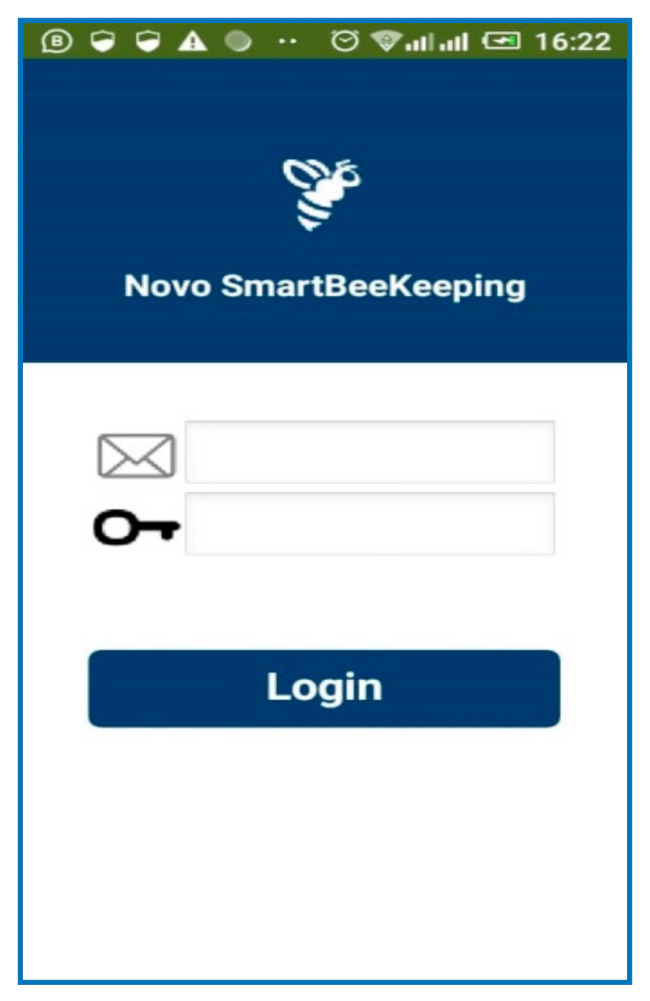
Mobil application user interface for SBMaCS.

Figure 16 shows how users access the platform through the interface of a mobile phone. They can regulate the temperature and the humidity of bees inside the hive through the application on their mobile phone and make decisions from where they are located. SBMaCS will help all beekeepers to implement smart bee farming with higher production.

**Figure 17:**
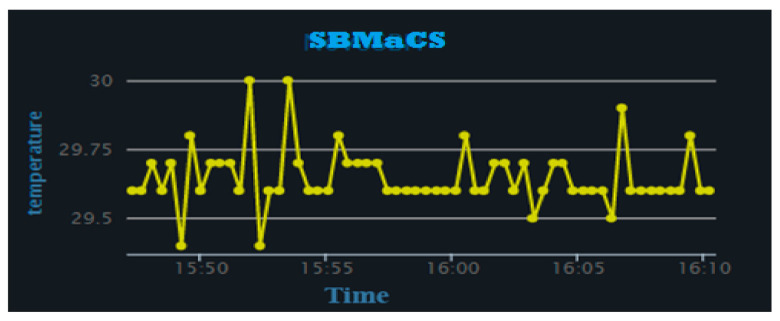
Temperature vs. time.

Figure 17 shows the plotted temperature variations over a period of time inside the beehive. The developed SBMaCS helps to regulate the temperature close enough between 30 and 36 °C, as it the favorable condition for colonies. The graph (Figure 17) clearly shows how the system keeps the temperature between the said range: at the minimum point of temperature, the system automatically heats the hive until it falls back in the range of the temperature favorable for bee colonies.

**Figure 18:**
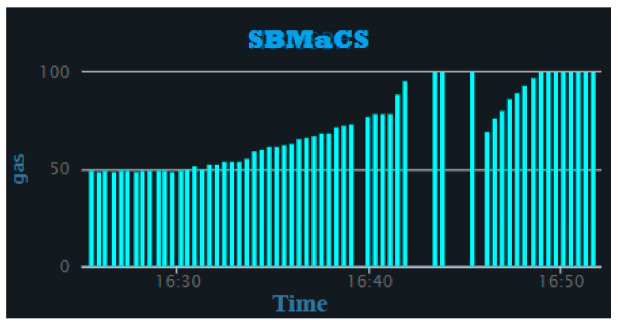
Gas vs. time.

Figure 18 shows the gas detection track of the beehive over a period of time, where the bars are rising from 0 to 50, which is the normal threshold value of the MQ-2 gas sensor. The bar values rising above 50 on the graphs only appear when any fragrant gases harmful to bees and colonies have been detected.

**Figure 19:**
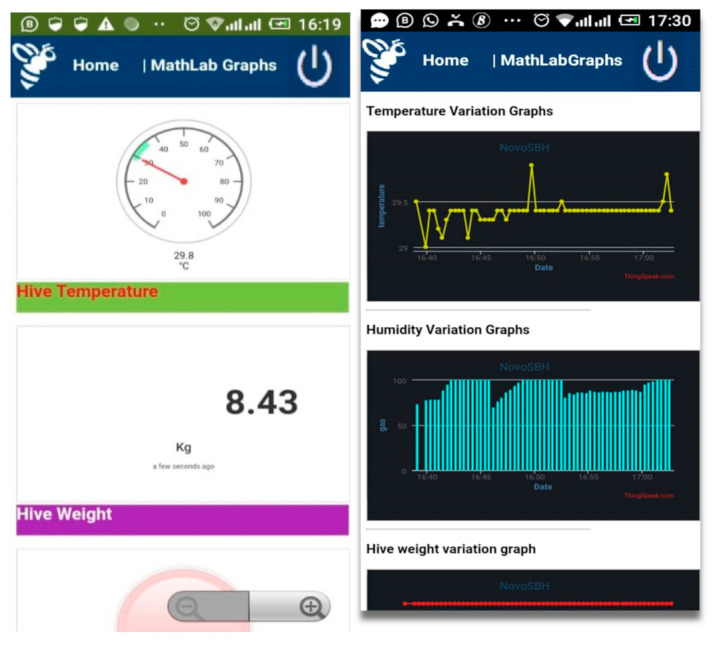
Different graphs of humidity, temperature, and weight.

**Figure 20:**
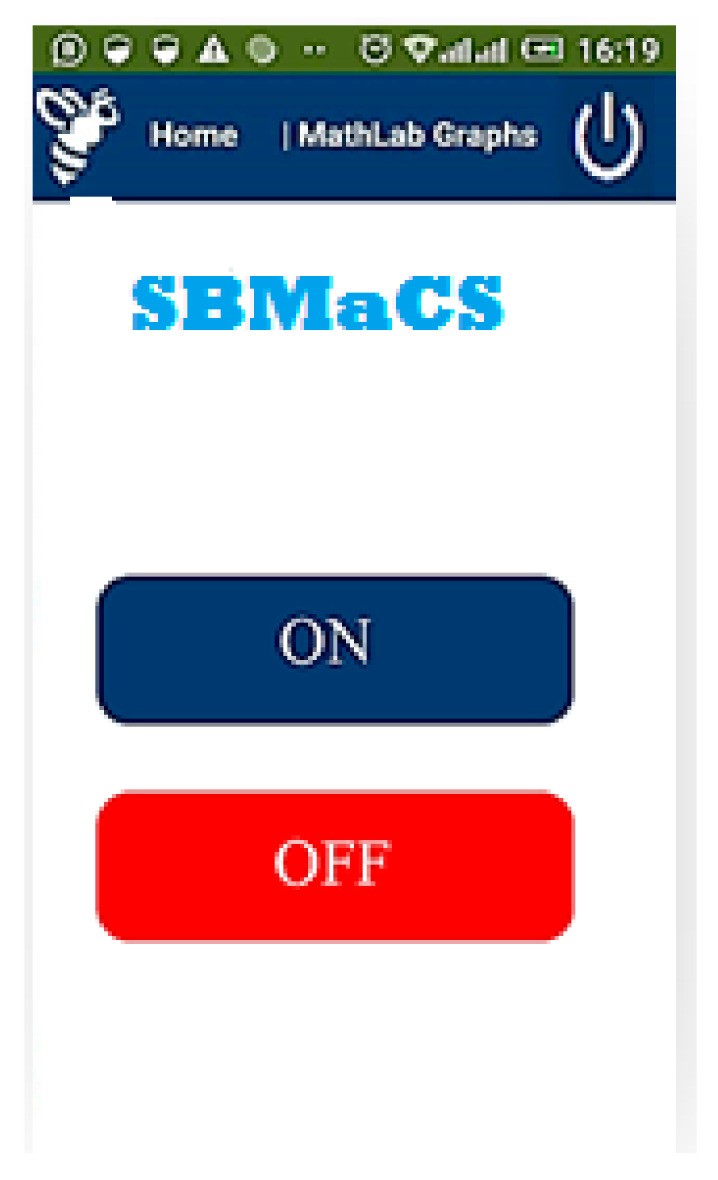
Graph of shutting down the system.

Figure 19 shows that all parameters of the beehive, such as humidity, temperature, weight of honey, gas provided by bees inside the hive, and any motion around the apiary were remotely controlled through the mobile phone application. An SMS notification is sent to the farmer’s mobile phone if smoke is detected due to a forest fire or a local fire set by people around the apiary. Furthermore, the mobile application of the SBMaCS helps beekeepers to remotely monitor the weight of the hive through their mobile phones and helps beekeepers to remotely collect, analyze, and make decisions, as it is shown in Figure 20.

**Figure 21:**
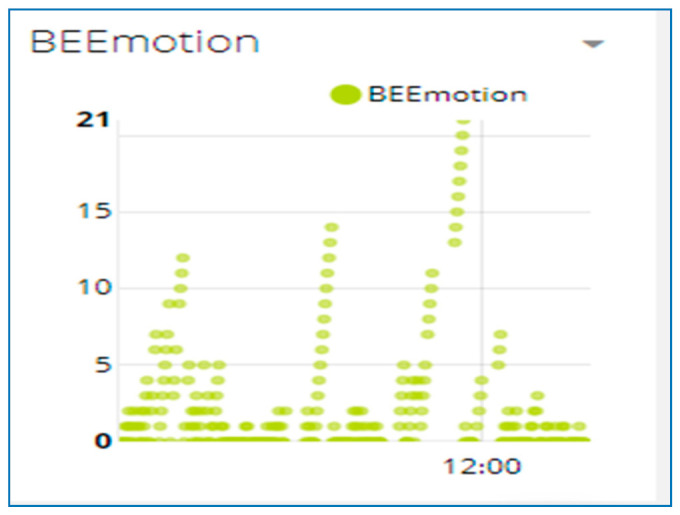
The graph of bees’ motion.

Figure 21 shows the bees’ motion. It shows the activity of the bees during the day and helps to compare the day-to-day activity of the colony. This gives an idea of when to minimize the opening of the entrance of the hive to prohibit the wax moths from entering the hive. It is seen that most wax moths enter the hive during the night, and if you take them out of the hive during the day (light) time, they fail to fly. Minimizing the opening of the entrance will minimize the risk of wax moths destroying the weak colony, and it minimizes the energy the bees spend to remove the wax eggs and larvae of the wax moths in the hive.

**Figure 22:**
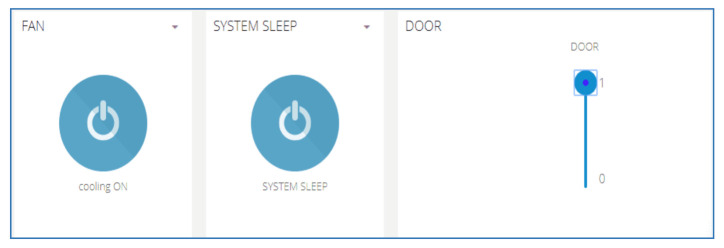
Control buttons in the mobile application of SBMaCS.

Figure 22 shows the various control buttons on the mobile application. The icon cooling ON in the mobile app allows an option for the farmers to switch the fan ON or OFF remotely when the temperature is high. The fan is also set to automatically switch on when the temperature goes above 32 °C and the fan automatically switches off when the temperature goes below 32 °C. The icon named SYSTEM SLEEP is the whole SBMaCS control. The system can be turned off to perform maintenance of hives or while harvesting the honey accumulated in the beehives. After completing all the activities in the apiary, the system can be switched on.

### 5.4. SBMaCS PCB Circuit Board

**Figure 23:**
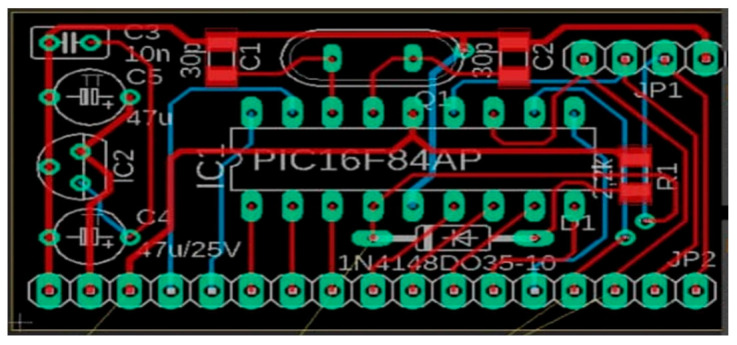
Design of the printed circuit board of the smart beehive mobile application system.

Figure 23 describes the design of a printed circuit board integrating the different sensors and actuators into a compact unit. This SBMaCS PCB is printed and various components are fabricated into a compact kit, which helps beekeepers to easily set their apiary. Specifications of this PCB board are described as follows:

- Arduino Uno with the following specification:
- Power: 3.3 V, 5 V, GND, analog pins: A0–A5 used to provide analog input in the range of 0–5 V. Input/output pins: digital pins 0–13 can be used as input or output pins. Serial: 0 (Rx), 1 (Tx) used to receive and transmit TTL serial data, and SPI: 10 (SS), 11 (MOSI), 12 (MISO), and 13 (SCK) used for SPI communication. Not only Arduino but also Rola radio node, with the following specifications:
  - Power supply: 3.7 V 14500 Lipo battery or 3.7–12 V DC power (VCC GND: 2PIN 2510-I Type connect), ATmega328P @ 8 MHz with 3.3 V logic/power, 3.3 V regulator with 500 mA peak current output. Hardware serial, hardware I2C, hardware SPI support, Pin #13 BLUE LED for general purpose blinking, 2 × analog inputs: A0 A12 mounting holes, 1 × PWM pins: D3 Size: 40 × 60 mm^2^. Reset button.
  - Finally, different sensors, with the specifications as follows: PIR sensor or digital camera is connected on pin A3, fan is connected on pin 7, relay is connected on pin 8, flame sensor is connected on pin 5, gas sensor is connected on pin A0, servo motor for automated ventilations is connected on Pin 6, DHT (temperature and humidity sensor) is connected on pin 4, buzzer is connected on pin 3, weight sensor is connected on pin 9, and GPS is connected on Pin 2.

## 6. Conclusions

In this paper, we designed a Smart Beehive Monitoring and Control System (SBMaCS) with energy harvesting technologies for self-powering SBMaCS. The developed system allows beekeepers to easily monitor and control the various parameters in their beehives. SBMaCS facilitates real-time weight measurement of the hive, ensures the security of the apiary, and helps to improve the health condition of the bees by controlling the beehive inside environment, thus maximizing the productivity even when the beekeeper is far from the beehives, through the use of mobile phones. Information from various sensors, such as temperature, humidity, weight, gas, and flame sensors, were collected and remotely regulated through the mobile phone application of the beekeepers or through an automated system connected on the Cloud. In addition, other embedded devices, such as electromagnetic heater, electronic fan, digital camera, and GPS to track the location of the hive, are also integrated within the SBMaCS. Self-powering the SBMaCS is achieved by using different energy harvesting technologies, such as piezoelectric energy harvesting based on the force of the hive, energy harvested from bees’ vibration and electromagnetic waves, and energy harvested from the surrounding environment where the hives are located. Furthermore, an integrated multi-sensing SBMaCS PCB has been developed. The usage and impact of this new SBMaCS in the sector of beekeeping in comparison with the existing traditional systems will be analyzed in the future research.

## Figures and Tables

**Table 1:** Power consumption of DHT11.

Parameters		Quantity
Resolution		8 bit
Power supply		3–5.5 V DC
Current supply	Standby	100–150 μA
	Average	0.2–1 mA
	Measuring	0.5–2.5 mA
Sampling period		1 s
Transistor logic (TTL) configurations power consumption		10 mW

**Table 2:** Power consumption by SBHMAS sensors and actuators.

Sensor	Active Current	Standby Current	Peripheral	Data Log Current	Transmission Current
MCU	0.029 μA	0.4995 μA	0.032 μA	0.0036 μA	N/A
DHT11	200 μA	40 μA	0	1 mA	N/A
MQ135	40 mA	2 mA	0	0.1–0.3 V	N/A
Piezoelectric module	0.8 μA	3.3 V	0	0	N/A
Flame sensor	15 mA	40 mA	0	0 to 5 V at 2 s	N/A
LoRa node	157 dB	45 μ A			20 dBm
PIR (motion sensor)	1.3 μA at 3 V	1.9 μA	N/A	100 μA	N/A
Thermoelectric heater	1.2 Watts at 5 V	0	N/A	N/A	N/A
Fan	0.14 A at 5 V	0	N/A	N/A	N/A
Digital camera	150 Μa	40 Μa	0	1 Ma	N/A
GPS	75 Μa	20 Μa	10 Ma	1 Ma	5 dbm

## Data Availability

Not applicable.
